# Cultural Perspectives on the Aftereffects of Combat Trauma: Review of a Community Study of Bedouin IDF Servicemen and Their Families

**DOI:** 10.5041/RMMJ.10205

**Published:** 2015-04-29

**Authors:** Yael Caspi, Ortal Slobodin, Ehud Klein

**Affiliations:** 1 Department of Psychiatry, Rambam Medical Health Care Center, Haifa, Israel;; 2 i-psy (intercultural psychiatry), Amsterdam, The Netherlands;; 3 The Bruce & Ruth Rappaport Faculty of Medicine, Technion-Israel Institute of Technology, Haifa, Israel

**Keywords:** Bedouins, combat trauma, IDF, PTSD

## Abstract

Combat trauma may affect servicemen from indigenous, traditional communities in ways that warrant special attention. The Bedouins, who enlist in the Israel Defense Forces (IDF) voluntarily, represent a unique, closed, collectivist cultural minority, potentially in a predicament in light of ongoing sociopolitical events. This paper summarizes findings and lessons learned from a community study of Bedouin IDF servicemen and their families residing in Israel’s Western Galilee. This is the only research endeavor to have addressed trauma exposure and posttraumatic reactions in this community. The sampling strategies and interview schedule were designed in consideration of participation barriers typical of hard-to-reach populations. Data collection followed an extended phase of liaising with key informants and building trust. Study limitations are discussed in terms of the challenges presented by this type of research. Interviews conducted with 317 men, 129 wives, and 67 mothers revealed high levels of trauma exposure and posttraumatic stress disorder (PTSD) in the men, and related distress in wives and mothers, but not in the children. The role of aggression in mediating the impact of PTSD and concepts such as shame, the loss of personal resources, and beliefs about retribution are highlighted as key issues for a culturally relevant understanding of traumatized indigenous communities.

## INTRODUCTION

The trauma of war tends not to wither when the fighting ceases. The direct impact on the soldiers, conveyed through the many faces of the post-traumatic distress, has now been studied long enough to warrant the change introduced by DSM-5.^1^ We have come a long way from the transient nature of the *Gross Stress Reaction* of DSM-I^2^ and the *Reaction to Extreme Stress* of DSM-II;^3^ the modified definition of posttraumatic stress disorder (PTSD) includes the understanding that exposure to severe trauma may result in persistent, exaggerated, and distorted negative beliefs and cognitions; persistent, negative emotional states, such as guilt, anger, shame, or fear; and persistent detachment and inability to experience positive emotions, such as love.^1^ In other words, these tenacious alterations in relationships to self and others represent what patients often describe as being lost to themselves.

The vicarious effects on the families of war veterans have also become widely recognized. Different studies in various countries concur that partners of veterans living with PTSD suffer from a range of mental health concerns,^4^ including symptoms of secondary traumatic stress and care-giver burden.^5^ Likewise, parental PTSD was also shown across multiple studies to be associated with child distress and behavioral problems.^6^

The body of knowledge on trauma and culture, accumulated primarily from studies of refugee groups from non-Western backgrounds, has revealed the challenges involved in the cultural translation and relevance of the traumatic stress concepts.^7^ However, the specific issues pertaining to military service of indigenous people, such as the Native American veterans,^8^ the Canadian First Nations,^9^ or the Australian Aborigines,^10^ have seldom been explored in spite of continuous participation in conflicts, pre-dating World War I. Little is known about exposure to combat trauma and its impact in these groups, who ordinarily come from disenfranchised, segregated communities with complicated and ambivalent relationships to the majority society regarding integration, loyalty, assimilation, and belonging.^8^

Israel, with its ethnic and religious diversity, provides a unique field of study for cross-cultural differences. Moreover, the Israel Defense Forces (IDF) (often considered to be the “melting pot” of Israeli society) includes, alongside the Jewish majority, soldiers and servicemen of Arab ethnicity, primarily from the Druze and Bedouin minority communities. While military service for the Druze, like their Jewish counterparts, is compulsory, the Bedouins join the IDF voluntarily. Given the unsteady and erratic nature of events in the Middle East, these closed, traditional, and understudied cultural groups find themselves periodically in the midst of public expressions of ethnic/religious tensions^11^ and anti-Arab sentiments.^12^

In this paper we provide an overview of findings and a summary of lessons learned from a community study of Bedouin servicemen and their families, residents of a large Bedouin village in Israel’s Western Galilee. The study was conducted in order to document the types and degree of trauma exposure among Bedouin servicemen, and to assess the impact of trauma on the emotional and physical health, functioning, and health services utilization of the servicemen themselves and of their family members. The study continued for over 3 years, during which we had the privilege to bear witness to multiple stories of severe combat trauma that were never disclosed by these men before. We were also privy to the voices of the women who often carry the brunt of the transformation in their husbands and sons.

Previous publications described specific findings about the servicemen, their wives, mothers, and children.^13^–^16^ The aim of the current report is to describe these different yet associated perspectives and present an integrated understanding of the complex individual, familial, and social costs associated with trauma exposure in this cultural minority group. Combined with anecdotal data from the interviews and the authors’ vast clinical experience with members of the Bedouin community, findings will be discussed in terms of several major themes that may be significant for culturally relevant interventions.

### Bedouins in Northern Israel

The Bedouins, of Arab ethnicity and Muslim faith, are a minority within the Arab minority in Israel, with distinctive Arabic “Bedawi” dialect and customs. Bedouin tribes in the northern parts of Israel originated primarily from the Syrian Desert and relinquished the nomadic lifestyle for permanent settlements before the state of Israel was established.^17^ Preserving a predominantly traditional lifestyle in the midst of growing progressive Western influences, the Bedouin villages maintain a tribal social structure and are segregated by choice from neighboring Muslim and Christian Arab communities. Marriages take place mostly within the extended family, and women retain traditional roles alongside growing interest in education and gainful employment.

Alliance with the Jewish military forces dates back to the struggle for Israel’s independence.^18^ Bedouin men, including those from tribes in southern Israel, volunteer for service in the IDF, primarily in military combat units. Much like the indigenous soldiers of North America,^8^ they have become legendary as knowledgeable, well-trained trackers, scouts, and light infantry.^18^,^19^

Several factors may cause Bedouin servicemen to be susceptible to the aftereffects of combat trauma exposure. These include the change in the nature of military activities that over the past several years have been primarily centered in the densely populated West Bank and Gaza. Although not openly discussed, it is presumed that the shared ethnicity and faith with the Palestinians may create dilemmas for Bedouin soldiers, as previously observed in minority soldiers deployed to conflict areas with high probability for civilian casualties.^20^ Concurrently, Muslim religiosity has been on the rise in these otherwise secular Bedouin communities, representing an unsympathetic attitude towards the voluntary enlistment in the IDF combat units.^21^ Discharge from the military is often accompanied by unemployment, loss of social and financial resources, and encounters with the regretfully widespread racial inequalities.^22^ In addition, due to the tribal structure of these societies, the loss or severe injury of a single soldier simultaneously affects multiple families. Added to the fact that cultural codes dictate suppression of personal grief and that communal rituals of remembrance are not customary,^23^ the impact on the community as a whole can only be surmised. Finally, once trauma-related distress becomes symptomatic, its identification as emotional problems requiring professional intervention is considerably delayed due to cultural barriers.^24^,^25^

## METHODS

### Setting

The second Palestinian Uprising (*Intifada*) in the West Bank and Gaza started in September 2000 and lasted for approximately 4 years. The Bedouin Desert Reconnaissance Battalion (Hebrew: *Gadsar*), which was founded in 1987, took a central part in the heavy fighting in the Gaza Strip. The battalion suffered multiple casualties and was commended for combat excellence by the Southern Command General. During those years, a few Bedouin veterans were admitted to the Department of Psychiatry at the Rambam Medical Health Center with unusual posttraumatic manifestations that generated a myriad of diagnoses, including PTSD with psychotic features, schizoaffective, adjustment, and personality disorders (Caspi, personal communication). Although attention to culture-specific manifestations of trauma-related distress and to reservations about the cross-cultural validity of the formal PTSD criteria has grown,^7^,^26^ information relevant to the unique experience and predicament of Israeli Bedouin soldiers simply did not exist.

With the purpose of exploring the type and degree of trauma exposure and its sequel among Bedouin servicemen and their families, the *Bedouin Community Outreach Project* was launched in 2003, in a village known for its high rate of military enlistment. This was the first such endeavor to have ever been attempted in the Bedouin community.

### Recruitment

Following nearly a whole year of forming relationships with leading members of the community who served as key informants for the study, door-to-door recruitment was carried out, aided by local recruiters representing the village’s five large tribal families. A detailed description of the study’s procedure is provided elsewhere.^13^ It is, however, important to stress that given that this community falls in the category of hard-to-reach populations, defined as members of socioeconomically disadvantaged groups,^27^ the sampling method (including some of the questions utilized) was designed in such a way as to curtail the anticipated barriers to consent and participation.

Recruitment, continuing over a period of 19 months, resulted in the identification of 372 men, of whom 348 (94%) agreed to participate and 317 (91%) completed the interviews. In consideration of cultural conventions, permission to interview the women was requested initially from the men. Interviews were completed with 129 wives and 67 mothers (for those participants who were unmarried). Interviews were conducted in Hebrew with the men due to their preference and language proficiency, and in Arabic with the women. An interview schedule, consisting of all instructions and questions, including self-rating scales, was translated into Arabic and back-translated into Hebrew to ensure item accuracy and relevance. Questions were read out loud, and responses were recorded by the interviewers. Interviews took place in the home, except for a few men who preferred to meet elsewhere.

### Participants

The 317 male participants averaged 30.4 years of age (SD 8.4), ranging from 18 to 62 years of age, were predominantly married (57.1%) or single (41.3%), with a mean of 11.6 (SD 2.3) years of schooling. More than half (58%) had children, with a mean of three live births (SD 2.5) and as many as 12 children. Nearly half (48.6%) described themselves as traditional and half (47.6%) as secular, with only a few (3.8%) as religious. Total time in service ranged between 1 month and 29 years, with a mean of 70.4 months—approximately 6 years (SD 76.4). Most participants (62%) were in service less than 5 years, 19% between 5 and 10 years, and 19% between 11 and 29 years. The majority (74.5%) served in combat positions (in descending order, as trackers, in the infantry, in specialized units trained in urban fighting, and in the border police). The rest were enlisted with the civilian police or in other non-combat units (e.g. the education or transportation corps). Those serving in combat units averaged 4 years more in service than those serving in non-combat units (*t*=6.92, df=241, *P*<0.001). At the time of the interviews 42% were still in service. Of the 183 discharged, 70% were discharged prior to the beginning of the study, with 14 men discharged for at least 10 years, and the rest between a few months and 9 years. Of those discharged, 38% were unemployed. Of all respondents, 12% were receiving benefits for some service-related disability.

The 196 women interviewed were related to 221 men, representing 70% of all male study participants. The majority of the women (*n*=179, 91.3%) were related to a single male participant, either husband or son, cohabiting in the same household. Of the 17 women related to more than one participant, 12 had 2–3 sons in the study and 5 had a husband in addition to 1 or more sons. For the purposes of statistical analyses, a single man was paired with each of these 17 women, according to a few systematic selection rules described elsewhere.^15^

### Measures

*Background variables* included age, years of schooling, duration of service, position in the military, and self-report of current financial status, rated on an ordinal scale from “excellent” (1) to “poor” (5). A binary variable was created by combining replies of “fair” and “poor” to indicate poor financial status (1) versus replies of “good,” “very good,” and “excellent” (0).

*Traumatic and stressful experiences* were drawn from a list of 13 events corresponding to the broader definition of DSM-IV-TR Criterion A1.^28^ These also included specific questions to the men about life-threatening events during military service and to the women about having been intimately touched against their will. An examination of the events described by the men was performed to ensure conformity to a strict definition of trauma, requiring actual presence at the site of the event.

*Structured Clinical Interview for Axis I DSM-IV Disorders* (SCID),^29^ Hebrew translation,^30^ was used to diagnose the men only.

*The PTSD symptom severity* was assessed by the Screen for Posttraumatic Stress Symptoms (SPTSS).^31^ This brief, 17-item, self-report instrument is rated on a scale from “never” (0) to “always” (10) in response to an enquiry about the frequency of the symptom during the past two weeks. Not keyed to a single event, the performance of the SPTSS in the men’s subsample indicated equal sensitivity and specificity rates of 89% vis-à-vis a SCID diagnosis of PTSD, at a cut point of 5.50.^32^ Internal reliability for both the men and women subsamples was very good (α=0.94).^14^

*Depression and anxiety symptoms* were assessed by the 25-item Hopkins Symptom Checklist (HSCL-25).^33^ This checklist consists of 10 items describing anxiety and 15 items describing depression (each rated on a 1–4 Likert scale). A cut-off score of ≥1.75 on the scale’s total mean score has been widely shown to indicate significant emotional distress and psychopathology in several cross-cultural settings,^34^ primarily depressive states in need of treatment.^35^ Consequently, HSCL-25 total scores in this study are reported as measures of depression symptom severity. The HSCL-25 has been translated and validated for Arabic-speaking populations,^36^ and it successfully detected psychiatric caseness in Palestinian primary health care patients in the Gaza Strip.^37^ Internal consistency was very good in the Bedouin men’s subsample (25 items, α=0.95; 10 anxiety items, α=0.91; 15 depression items, α=0.92), and good in the wives’ subsample (25 items, α=0.89; 10 anxiety items α=0.83; 15 depression items, α=0.80).^14^

*Mothers’ report of children’s problems.* Mothers were asked to report whether their children experienced any problems from a list including seven manifestations of emotional and behavioral difficulties: argumentative/conduct problems, concentration/attention problems, lying/concealing, stealing, anger outbursts, sadness/frequent crying, and loneliness. Responses were measured by a binary variable and reflected the presence of the problem in at least one of the children. In the statistical analysis the total number of problems was used. Mothers were also asked to report the frequency of school days missed in the past month, separately for children in elementary school and in high school. Responses ranged between “never” (0) and “very often” (5).

*Substance use*, including cigarettes, beer, wine, and hard liquor, was assessed only for the men. As suggested by the key informants, questions about drug use were avoided because alcohol is considered to be the substance of choice in this community, and due to concerns that inquiring about illegal activities may jeopardize honest replies.

*Physical health, health-related functioning, and utilization of health services* were assessed by eliciting information on somatic symptoms, self-perceptions of health and well-being, and self-perceived limitations in work, family, and daily functioning due to physical and emotional health (adapted from the SF-36).^38^

Feelings of guilt and shame were assessed with men only by four questions rated “not at all” (0) to “extremely” (10): Degree of guilt and remorse regarding service-related events; Degree of shame related to current functioning; Degree of shame related to others thinking there is something wrong with you; Degree that shame interferes with your ability to do things and act as you wish. Internal consistency was good (Cronbach’s α=0.82).

*Men’s aggression*, as reported by the women, was constructed as an index measure from three items describing behaviors that increase in level of volatility: 1) irritable, angry, or impatient reactions; 2) breaking things, slamming doors, or losing temper (verbally); 3) physical fights. The choice of these items was guided by concerns that women would refuse to answer more direct questions about husbands’ aggressive behavior in the home setting. The frequency of each item was rated on an ordinal scale from “never” (0) to “most of the time” (4). Scores on all three items were added to construct a continuous measure of men’s aggression in the past 6 months, ranging from 0 to 12. Internal consistency was fair (Cronbach’s α Wives=0.77, Mothers=0.57).

*The Conservation of Resources Evaluation* was used to measure the extent to which Bedouin servicemen had experienced loss of resources.^39^ Resources are divided into four categories: *Object resources* include physical objects linked to socioeconomic status; *Condition resources* facilitate the acquisition and protection of valued resources, such as seniority at work, or stable marriage; *Personal resources* refer to qualities that people possess, such as self-esteem, self-mastery, and sense of success; *Energy resources* may be exchanged for resources in the other three categories, such as money, health, and credit. In the Bedouin sample, our findings suggest that the loss of personal resources, rather than resources from the other categories, mediates the impact of traumatic exposure on the development of psychological symptoms, such as re-experiencing, arousal, depression, and anxiety.

The present study used 42 items from the questionnaire: 4 items of object resources, 14 items of condition resources, 15 items of personal resources, and 9 items of energy resources. The amount of resources was rated on a scale ranging from “not at all” (1) to “very much” (5), in reference to two points in time: before the traumatic event and in the present. For each item, a resource loss score was created by calculating the difference between the resource score before the traumatic event and the resource score in the present, with higher scores indicating more loss. In addition, a resource loss score was created for each of the four subscales (Object, Condition, Personal Characteristics, and Energy). Test-retest reliabilities of the scores, with participants rating losses and gains during the past year, ranged from *r*=0.64 to *r*=0.67 over a 2-week period.^40^ Internal reliability of the questionnaire in this study was α=0.96. Internal reliabilities of the four resources subscales were object resources, α=0.58; condition resources, α=0.90, personal resources, α=0.94; and energy resources, α=0.81.^41^

### Data Analyses

Analyses in this paper are based on the hypothesis that men with PTSD have more difficulties than men with other DSM diagnoses and men with no diagnosis at all, as have the women related to them. Analyses of the men’s data employed the three mutually exclusive diagnostic groups: PTSD, DSM diagnosis other than PTSD, and no diagnosis. Analyses of the mothers’ data employed only two diagnostic groups due to small subsamples sizes: any DSM diagnosis and no diagnosis (in sons). Comparisons were made on several background and outcome measures using adjusted analysis of variance models for continuous variables with Bonferroni *post-hoc* comparisons. Analyses were performed with SPSS version 21.0.

In order to provide a review of all the main findings from the Bedouin community study, findings presented below are also drawn from analyses described in previous papers, which are clearly referenced.

## FINDINGS

### The Impact of Trauma and PTSD

#### Bedouin Servicemen

Traumatic life experiences involving actual presence at the scene of the event, and including combat, severe car accidents and other accidents, terrorist attacks in civilian settings, and interpersonal violence, were reported by 238 (75.1%) respondents. An additional 22% reported extremely stressful events. Combat trauma was experienced by 214 men, 90% of all those traumatized. These experiences included comrades killed in action, caring for body parts, suicide bomber’s explosion, being trapped in ambush under incessant fire, and sustaining severe injuries during military action.

Posttraumatic stress disorder as defined by DSM-IV-TR^28^ was diagnosed in 46 men (14.5%; 20% of those traumatized), mostly co-morbid with major depression disorder and alcohol abuse. Forty men (12.6%) were diagnosed with other DSM diagnoses, mostly generalized anxiety and panic disorders. Table 1 shows the relationship of trauma exposure to diagnostic status in our sample, indicating the increased and significant likelihood of a DSM diagnosis, especially PTSD, given exposure to war trauma and severe car accidents. Nevertheless, a powerful finding of this study, described in an earlier publication of these data,^13^ was that PTSD, and not trauma exposure by itself, was the factor associated with significant impairment in emotional health, physical health, and functioning. Indeed, comparisons of traumatized servicemen with PTSD (*n*=46) to those exposed to trauma but without PTSD (*n*=192) and to those who did not experience trauma (*n*=79) yielded significant group effect on all symptom checklists, on daily consumption of cigarettes and alcohol, and on physical health complaints and utilization of health services. Those exposed to trauma but without PTSD appeared to form an intermediary group, but significant differences were noted primarily between those with PTSD and those not exposed to potentially traumatizing events.

**Table 1.** Trauma Exposure by DSM Diagnostic Group in 317 Bedouin IDF Servicemen.

	**DSM Diagnostic Groups**			
	**PTSD, *n* (%)**	**Other DSM Diagnosis, *n* (%)**	**No Diagnosis, *n* (%)**	**Chi-square, *P* value**
**Types of traumatic exposure^a^**	46 (14.5%)	40 (12.6%)	231 (72.9%)	
**War**	44 (95.7%)	30 (75.0%)	140 (60.6%)	22.65, *P*<0.001
**Car accidents**	17 (37.0%)	11 (27.5%)	38 (16.5%)	11.02, *P*<0.01
**Violence**	2 (4.3%)	3 (7.5%)	5 (2.2%)	3.43, NS
**Other accidents**	2 (4.3%)	0 (0%)	7 (3.0%)	0.45, NS
**Terror attacks**	3 (6.5%)	1 (2.5%)	10 (4.3%)	0.83, NS
**No exposure**	0 (0%)	7 (17.5%)	72 (31.2%)	21.26, *P*<0.001

a Trauma categories are not mutually exclusive.

DSM, Diagnostic and Statistical Manual of Mental Disorders; PTSD, Posttraumatic Stress Disorder.

As shown in Table 2, comparisons of men’s variables across the three diagnostic groups—PTSD, other DSM disorders, and no disorders—provides a supporting perspective on these findings. While there were no differences in age, years of schooling, or number of children, significant differences were noted in level of trauma exposure, symptoms of PTSD and depression, physical complaints, self-reported poor health, and angry outbursts. Those with PTSD were always more severely affected than those with other DSM disorders, who in turn were worse than men with no disorders at all.

**Table 2.** Overview of Study Participants by Men’s DSM Diagnostic Group.^a^

	Men (*n*=137)			Wives (*n*=129)			Mothers (*n*=67)	
		
	PTSD (*n*=46)	Other DSM Dx (*n*=40)	No Dx (*n*=231)	Husbands w PTSD (*n*=26)	Husbands w Other DSM Dx (*n*=19)	Husbands w No Dx (*n*=84)	Sons w Any DSM Dx (*n*=18)	Sons w No Dx (*n*=49)
Age	32.50 ± 9.39	30.14 ± 6.90	29.93 ± 8.44	31.44 ± 9.93	31.79 ± 7.16	31.10 ± 7.70	52.39 ± 6.60	51.38 ± 8.57
Years of schooling	10.83 ± 1.88	11.35 ± 1.93	11.77 ± 2.44^*^	9.56 ± 4.09	10.68 ± 2.36	10.99 ± 2.93	4.00 ± 3.89	3.65 ± 3.86
Number of births	2.43 ± 2.67	1.90 ± 2.24	1.85 ± 2.54	3.23 ± 2.79	3.95 ± 2.50	3.42 ± 2.38	8.78 ± 2.82	8.63 ± 2.73
Employed	20 (43.5%)	29 (72.5%)	199 (86.1%)^***^	3 (11.5%)	2 (10.5%)	19 (22.6%)	0 (0%)	1 (2%)
Low financial status ^b^	-	-	-	18 (69.2%)	8 (42.1%)	34 (40.5%)^*^	13 (72.2%)	33 (68.8%)
Sum trauma events	4.26 ± 1.34	3.78 ± 1.16	2.98 ± 1.49^***^	2.31 ± 1.57	2.42 ± 1.12	1.58 ± 1.29^**^	2.33 ± 1.28	2.27 ± 1.58
SPTSS average	7.41 ± 1.72	4.66 ± 2.61	1.93 ± 1.81^***^	2.95 ± 2.08	2.05 ± 1.73	1.65 ± 1.47^**^	2.68 ± 1.89	2.72 ± 2.03
HSCL average	2.67 ± 0.62	1.90 ± 0.58	1.34 ± 0.33^***^	2.04 ± 0.41	1.71 ± 0.42	1.65 ± 0.44^***^	1.99 ± 0.48	2.02 ± 0.49
Sum somatic symptoms	5.28 ± 3.05	2.57 ± 2.49	0.80 ± 1.46^***^	4.31 ± 2.69	2.53 ± 1.61	2.48 ± 2.08^**^	6.11 ± 2.17	5.18 ± 2.15
Fair/not good/poor health	41 (89.1%)	14 (35.0%)	61 (26.4%)^***^	4 (15.4%)	1 (5.3%)	5 (6.0%)	10 (55.6%)	19 (39.6%)
Man’s angry outbursts	44 (95.7%)	30 (75.0%)	101 (43.7%)^***^	23 (88.5%)	12 (63.2%)	33 (39.3%)^***^	6 (33.3%)	22 (45.8%)
Family problems ^b^	-	-	-	12 (46.2%)	2 (10.5%)	10 (11.9%)^***^	3 (16.7%)	9 (18.4%)

Values are given as Mean ± SD, or *n* (%).

a Pearson’s *r*, chi-square, and *F* statistics are available from the authors by request.

b These questions were only included in the women’s interview.

* *P*<0.05;

** *P*<0.01;

*** *P*<0.001.

DSM, Diagnostic and Statistical Manual of Mental Disorders; Dx, Diagnosis; HSCL, Hopkins Symptom Checklist-25; PTSD, posttraumatic stress disorder; SPTSS, Screen for Posttraumatic Stress Symptoms; w, with.

As previously reported,^15^ PTSD rather than trauma exposure was also shown to be associated with smoking more cigarettes and drinking more beers, poor and worsening health status, reports of frequent illnesses, and more conditions diagnosed by a physician such as ulcers, asthma, high blood pressure, and ruptured discs.^13^ While participants with PTSD were also more likely to utilize primary health care services, psychiatric care was very infrequent; fewer than half of those diagnosed with PTSD had ever sought mental health services.^15^

Our data also show that in comparison to men with other DSM disorders and men with no disorders, those with PTSD were more likely to report that shame interfered with their daily life (chi-square=91.68, *P*<0.001), more likely to report feeling guilt and remorse regarding service-related events (chi-square=37.75, *P*<0.001), shame about their current functioning (chi-square=95.22, *P*<0.001), and shame about others’ perceptions of them (chi-square=88.86, *P*<0.001). In addition, controlling for the effect of men’s diagnostic status, shame related to others’ perception was significantly associated with multiple health and functioning items, such as increased likelihood for poor or bad health status, more frequent occurrence of illness and more frequent visits to the primary care clinic, increased impairment in self-care, social relations, familial role, and daily functioning.

#### Wives and Mothers of Bedouin Servicemen

The 129 wives averaged 31.27 years of age (SD 8.06), with 10.66 years of schooling (SD 3.15) and an average of 3.46 births (SD 2.47). Nearly 47% reported fair/poor financial status, and 8% reported fair/poor health status. The 67 mothers averaged 51.5 years of age (SD 8.04), with 3.75 (SD 3.84) years of schooling and an average of 8.67 births (SD 2.73). Nearly 70% reported fair/poor financial status, and 44% reported fair/poor health status.

As shown in Table 2, the 129 wives were similar in age, years of schooling, number of births, and employment status whether their husbands were diagnosed with PTSD, other DSM disorders, or had none at all. However, those with husbands suffering from PTSD were experiencing significantly lower financial status, more stressful events, increased symptoms of post-trauma and depression, and more somatic symptoms. Wives of men with PTSD also reported significantly more men’s angry outbursts and family problems. Those whose husbands had other disorders were clearly an intermediate group, with differences from the PTSD or the no-diagnosis groups not always reaching statistical significance levels.

In a paired subset of our sample,^14^ composed of 129 couples, wives averaged 31.27 (SD 8.06) years of age with 10.66 (SD 3.15) years of education and an average of 3.46 (SD 2.47) births. Poor financial status was reported by 46.5%, and 44.2% reported frequent visits to the primary care clinic in spite of only 7.8% reporting poor health status. Findings indicated that wives of men with PTSD exhibited significantly more severe symptoms of posttraumatic stress and depression and more somatic complaints than wives of men with other DSM disorders or with no diagnosis. An important finding was that husbands’ aggression (reported by 52.7% of the wives) fully mediated the relationship between husbands’ posttraumatic symptomatology and wives’ emotional and somatic problems.^14^

The 67 mothers had 6 sons with PTSD, 12 sons with other DSM disorders, and 49 sons with no diagnosis. As a result, a joined group was formed of any DSM disorders. As shown in Table 2, analyses indicated that the mothers were similar across all the variables disregarding their sons’ diagnostic status. In a paired subsample of the 67 Bedouin mothers and their sons, the mean age was 51.65 (SD 8.04), with only 3.75 (SD 3.84) years of schooling, and an average of 8.67 (SD 2.73) births.^15^ Nearly 70% reported poor financial status, and 44% reported poor health. Physician-diagnosed illnesses were reported by 64.2%, and 65.7% visited the primary care clinic frequently. Hierarchical regression analyses indicated that, with no exceptions and in contrast to the wives, sons’ diagnostic status was unrelated to mothers’ emotional and somatic distress. Rather, mothers’ poor financial status was the most significant and consistent variable that was related to mothers’ well-being. However, mothers’ utilization of primary health care services was significantly and positively associated with sons’ aggression, reported by 41.8% of the mothers.^15^

#### The Children of Bedouin Servicemen

In order to address the question of intergenerational transmission of trauma and the ways by which the level of fathers’ posttraumatic symptoms was associated with mothers’ report of children’s problems, a subset of the larger sample was selected that included 112 couples with a total of 411 children.^16^ Half of the children (50.1%) were males, with a total mean age of 9.57 (SD 6.43), ranging from 2 months to 30 years. Data on age were missing for two children, and data on gender were missing for three children.

Findings indicated that the levels of father’s posttraumatic symptoms, as well as depression and anxiety, were positively associated with mother’s depression and anxiety symptoms, but not with maternal reports about children’s variables. Among the sociodemographic and clinical variables, only financial status and father’s education were associated with the study variables, while the number of children in the family was not. In contrast, increased levels of mother’s depression, anxiety, and post-traumatic symptoms were associated with more reports of children’s problems and higher number of missed school days, for both elementary and high school children.

## DISCUSSION

The Bedouin community study is the first methodical examination of the effects of trauma exposure and posttraumatic disorder in a sample of Bedouin servicemen and their families. In previous publications we discussed the findings according to the different subgroups within our sample. In this paper we wish to highlight several themes that are not only at the core of the predicament of this community but, in our view, are also pertinent to other traditional, indigenous groups involved in military activities.

### The Bedouin Family in the Shadow of PTSD

Living with and caring for veterans suffering from combat PTSD was shown to result in identifiable emotional distress^5^ and a range of physical and mental health concerns among spouses.^42^ Studies repeatedly linked PTSD symptomatology rather than trauma exposure by itself with intimate partner violence and family aggression.^43^ Information on the impact of veterans’ PTSD on families from non-Western communities was not available until this study.

Our clinical experience with the Bedouin families whose husbands or sons suffer from combat-related PTSD highlights the plight of the wives. Commonly, having moved after marriage from their own family into or close to the husband’s extended family household, these women must cope with role reversal, lack of support, full responsibility for the family, deteriorating economic status, and significant loss of their previous life. Similar consequences have been described among Israeli Jewish wives.^44^ However, given that the Bedouin cultural and social codes prohibit the direct expression of emotional discomfort and the fear of stigma if husband’s impairment is exposed, Bedouin wives often experience complete isolation. The lack of familiarity with the concepts of trauma and PTSD adds to the sense of marital failure that is further concealed from others, including and even especially their own family members.

The differences noted between the impact on wives and mothers were illuminated by conversations with Bedouin community members who described the typical Bedouin mother as not necessarily supportive of her son’s military enlistment nor aware of what military service entails. Needless to say, if the son is beginning to show signs of PTSD, the inevitable deterioration in his conduct towards family members at home is perceived as lack of respect towards the parents and a breakdown of the family system, and is not at all associated with the son’s military service. In one such example, the elderly mother of a suicidal and aggressive veteran with severe undiagnosed PTSD, left her family home and moved in with her own elderly father, a drastic and culturally improper behavior. Years later, when the son was diagnosed, she expressed deep regret for her lack of understanding (Caspi, personal communication).

The impact on children in these Bedouin households was addressed by our study only in a limited manner,^16^ and more systematic evaluation of the potential risks should be undertaken. One of the offered explanations for the lack of association between father’s PTSD and children’s well-being, suggested in other studies, is that additional family members step in to offset the impact of the veteran’s PTSD.^45^ This may be especially relevant for the tribal Bedouin family where the extended family plays an important role in the child’s upbringing.^46^ Clinical experience with Bedouin IDF members with PTSD suggests that, oftentimes, an older brother or brother-in-law becomes more involved with the veteran’s children, thus protecting them, to some degree, from their father’s emotional condition (Caspi, personal communication). Wives, especially once aware of the meaning of PTSD, usually take an active role in minimizing triggers that may precipitate PTSD symptoms in their husbands^47^ and assume even greater responsibility for the children.^48^

### Loss of Resources

Bedouin men with PTSD express a deep sense of loss that pertains to all aspects of their lives: from self-respect and their place in the family and in the community, to their professional development and economic security. In order to capture this aspect of the effect of exposure to combat trauma and PTSD, our study utilized the conservation of resources (COR) theory.^49^ This general stress theory suggests that individuals strive to obtain, retain, protect, and foster those things that they value. Loss of resources is considered the primary mechanism through which stressful and traumatic events impact individuals’ psychological health.

Given the strong tribal structure of the Bedouin society,^50^ and the emphasis on communal values (e.g. social reputation, recognition, and status) rather than individualistic ones (e.g. personal happiness or “having an interesting life”),^51^ the apparent importance of personal resources is surprising. However, traumatic experiences often involve feelings of guilt, worthlessness, shame, and personal failure,^52^ and these distortions in self-perception are viewed by several researchers as the core phenomenon of PTSD.^53^,^54^ Our clinical experience shows that the loss of personal resources such as self-worth, self-respect, and self-mastery and the inability to meet social and role expectations are especially damaging for Bedouin men suffering from PTSD. This may be the consequence of precisely those values of collectivist communities that place amplified importance on the way one is perceived by others.

### Shame

One of the changes introduced by the DSM-5 definition of PTSD is the addition of Criterion D “Negative alterations in cognitions and mood that are associated with the traumatic event(s)” and, within it, the inclusion of persistent and exaggerated negative expectations, persistent distorted blame, or pervasive negative emotional state such as fear, horror, anger, guilt, or shame. Posttraumatic shame has been identified as an important dimension of posttraumatic syndromes^55^,^56^ and suggested as the missing link to the unraveling of complex trauma reactions in non-Western communities.^57^

As we learned from focus groups and Bedouin key informants, the Bedouin cultural and social codes encourage suppression of emotions in men—the adjectives used were “robotic” and “frozen.” Expressed emotional pain is a sign of character weakness and pathological behavior. However, shame operates as an inner warning signal: the expectation of shameful feelings is a threat to the sense of integrated self and triggers automatic responses such as flight, anger, or concealment. The fact that these posttraumatic symptoms cannot be masked is distressful for all who suffer from it, and exceptionally so for patients of Arab ethnicity.^58^ The sensations that accompany the experience of shame become traumatic triggers by evoking feelings of humiliation and powerlessness that are simultaneously a reflection of the current disability and a cue to re-experiencing the traumatic event. Given that there is little control over the “when” and “where” these emotions erupt, shame and alienation spiral, leading to further shame, exacerbated posttraumatic symptoms, and isolation.^59^

Most non-Western communities emphasize collectivist cultural values such as strong ties between individuals, group solidarity, emotional interdependence, traditionalism, and a collective identity, as opposed to independence and individual autonomy.^60^ In the face of trauma, the interconnectedness of family members may have negative consequences, not only because a large circle of extended family members are affected,^61^ but also due the reduced tolerance for deviations from the norm. The complex Bedouin values of honor and independence are firmly connected to self-control. Failings or weaknesses disqualify the person. Men especially thus find themselves in a dependent and vulnerable position.^62^ Consequently, and given the lack of familiarity in this community with the concept of trauma and posttraumatic reactions, the damage caused by PTSD to the Bedouin man’s sense of self-worth is devastating.

### Retribution

Another important issue that requires further exploration relates to the concept of retribution.^63^ Central to the Bedouin code of honor and justice, the right to retribution dictates that when a life is taken, even if under justified circumstances, the person responsible cannot get away without penalty. Many of our participants with PTSD disclosed thoughts about the cross-generational weight of their actions during combat—“If I don’t pay, my children will pay. If not them, then their children ... until it [the score] is settled.” Moreover, the right to retribution is shared with the enemy—the mother whose armed son was killed at close range, the terrorist whose attack was intercepted but got away—the threat is tangible and present.

The cultural suitability of the more researched therapeutic interventions for this community has not yet been established. Cultural belief systems are powerful but neither completely inflexible nor unchanging, as numerous anthropological studies around the world have shown. It would be important to investigate innovative but culturally acceptable ways in which this potent belief about retribution and its possible intergenerational transmission can be addressed in interventions and then to study their effectiveness.

### Study Limitations

To successfully conduct a research project within a community as closed and traditional and as proud and sensitive as the Bedouin community requires extensive preparation, flexibility, acceptance, and a genuine respect for differences. There are many methodological limitations in the Bedouin community study. We attempt to present them here through the lens of studying hard-to-reach populations. We believe that the care we took in approaching and engaging the community in this research study bred trust and a willingness to take part in an opportunity to address a very painful and hidden problem.

The primary limitation of this study stems from its sampling method. A comprehensive list of all village residents enlisted in or discharged from the IDF was unavailable, and attempts to get one were unsuccessful. A random sample, necessary for a valid generalization of findings, could therefore not be drawn. The sampling strategies employed drew from aspects of snowball, chain-referral sampling,^64^,^65^ employed with hard-to-reach groups. We used recruiters from within all five tribal families in order to avoid the tensions and historical feuds that openly exist among some of them. The fact that 94% of those approached agreed to participate, and that the 317 who completed the study represent 85% of all approached, is a response rate that exceeded our expectations. Nevertheless, it is possible that more IDF households were not identified and that those who refused were suffering from higher rates of trauma and disability. Therefore, the degree to which this sample is representative of all IDF members in this specific community and in other Bedouin villages in the Galilee remains unknown.

High rates of trauma exposure were experienced by this sample, with three of four men exposed to at least one type of traumatic event of sufficient severity potentially to cause PTSD, and war trauma reported by nearly 70% of the total sample and by 90% of those traumatized. A formal diagnosis of PTSD was identified in 14.5% of the men, 20% of those traumatized.^13^ The lack of a comparison group of Jewish soldiers who participated in the same or similar combat activities does not allow for a valid evaluation of the true meaning of these observed rates. Nevertheless, our findings seem to be comparable to the estimated rates of PTSD for Operation Enduring Freedom and Operation Iraqi Freedom (OEF/OIF) veterans reported to range between 8.5% and 15.8%.^66^–^68^

The cross-sectional design of this study limits our ability to explore the observed relationships among the variables, specifically the strong associations between a PTSD diagnosis and all the variables indicating impairment in emotional and physical health and functioning. However, much has already been documented regarding the association of PTSD symptoms with increased onset of physician-diagnosed disorders in veterans,^69^ as well as lower ratings of general health, more sick-call visits, more missed workdays, more physical symptoms, and high somatic symptom severity.^70^ Our clinical experience also supports the impression that the symptoms of PTSD, especially in this cultural group, instigate a cycle of deteriorating self-image and self-care that exacerbates the PTSD symptoms and results in increased levels of tension and additional somatic complications.

Third, data on traumatic and stressful life events were lacking in two ways: information about the sequencing of the events or their proximity to the onset of symptoms was not collected. It is also important to note that when asked during the interview about the temporal order of events, it appeared to be difficult for participants to relate to a linear description, nor did it seem to be of importance to their story. Anecdotal data from the interviews suggested that functional impairment related to PTSD symptoms (especially avoidant behavior) was experienced primarily after discharge from service even when symptoms such as repeated nightmares or disturbing thoughts of the event were already present. Although indicative of latent and delayed posttraumatic response, systematic data to support it cannot be drawn from this study.

Finally, the variables measuring men’s aggression and children’s problems were not drawn from validated measures, but were suggested by our key informants. It is important to understand that, given the intrusive nature of the interview and the internalized and public stigma regarding mental health, there was no certainty that women would be permitted or willing to participate, not to mention to speak openly about negative feelings or difficult behaviors attributed to their husbands or sons. Therefore, several concessions in the choice of instruments and in the length of the interview were made.

## Abbreviations:

DSM: Diagnostic and Statistical Manual of Mental Disorders;
HSCL-25: Hopkins Symptom Checklist-25;
IDF: Israel Defense Forces;
PTSD: Posttraumatic Stress Disorder;
SCID: Structured Clinical Interview for Axis I DSMIV Disorders;
SPTSS: Screen for Posttraumatic Stress Symptoms.
